# Mercury

**Published:** 1872-06

**Authors:** 


### MERCURY,

In the form of calomel and corrosive sublimate, is medicinal and poisonous. The latter article, mixed with the white of eggs, is a favorite means of destroying bedbugs; and as such is found in bottles carelessly standing about in many houses; life has often been destroyed by this means. The presence of these articles in pills or liquid form is proven thus: if in the form of a pill, make a paste of it with water, put it on a well burnished piece of copper, add to it a little ether or iodide of potassium; stir it with a pointed penknife, and if there is mercury present, in small quantity, a white, silvery spot will be left on the copper. Swallowing corrosive sublimate causes burning in the throat, excessive pain in stomach and bowels, intense thirst, sick stomach and vomiting, diarrhœa, bloody stools, convulsions, and death. Swallow promptly the whites of several eggs; if not at hand stir a tablespoon or two of flour in half a pint of water, and drink it down instantly, and repeat every fifteen minutes until the symptoms abate; meanwhile, send for the nearest physician.

